# Trade-Offs and Driving Factors of Microbial Carbon and Nitrogen Use Efficiency in Typical Forest Ecosystems of Funiu Mountain

**DOI:** 10.3390/microorganisms14071580

**Published:** 2026-07-20

**Authors:** Yadong Xu, Yiran Lai, Luotong Zhao, Shujuan Guo, Tianfu Han

**Affiliations:** 1 School of Agriculture and Biomanufacturing, Zhengzhou University, Zhengzhou 450001, China; xyd2020@zzu.edu.cn (Y.X.); laiyiran0708@163.com (Y.L.); 2 Henan Funiu Mountain Biological and Ecological Environment Observatory Research Project, Zhengzhou 450001, China; 3 School of Life Sciences, Zhengzhou University, Zhengzhou 450001, China; 13783152637@163.com

**Keywords:** carbon use efficiency, nitrogen use efficiency, litter quality, extracellular enzyme activity, forest

## Abstract

Soil microbial carbon use efficiency (CUE) and nitrogen use efficiency (NUE) are fundamental parameters governing organic matter turnover in terrestrial ecosystems, yet how forest type-driven variation in litter quality propagates through the litter–soil–microbe continuum to regulate these efficiencies remains poorly resolved. Across three forest types in the Funiu Mountains, central China—a *Larix gmelinii* (LG) plantation, a *Quercus aliena* var. *acuteserrata* (QA) secondary forest, and a mixed *Quercus aliena* var. *acutiserrata* and *Pinus armandii* (QP) forest—we quantified litter chemistry, soil physicochemical properties, microbial biomass, extracellular enzyme activities, and microbial nutrient use efficiencies (MUE: NUE, and phosphorus use efficiency, PUE) derived from a modified saturation kinetics model. Principal coordinate analysis revealed significant multivariate differentiation among forest types across litter, soil, microbial biomass, and enzyme modules (Adonis R^2^ = 0.198–0.427; all *p* < 0.05). Compared with LG and QA, QP exhibited a pronounced stoichiometric imbalance: it supported the highest litter organic carbon and total nitrogen, the lowest lignin-to-cellulose ratio, the largest soil C and N pools (SOC and STN), and the greatest microbial biomass carbon (MBC). However, despite this resource-rich environment, microbial biomass C:N:P ratios exhibited constrained variation, while soil C:P (SCP) and N:P ratios (SNP) in QP reached extreme values (112.3 and 7.25, respectively), generating severe stoichiometric imbalance. Vector analysis indicated that all forests were under relative nitrogen limitation (vector angle < 45°), with QP showing the strongest limitation (41.6 ± 0.4°). Critically, QP exhibited the highest NUE (0.47 ± 0.03) but the lowest CUE (0.95 ± 0.01), and CUE and NUE were nearly perfectly negatively correlated across all sites (R = −0.98, *p* < 0.001). Random forest analysis identified extracellular enzyme stoichiometry as the dominant proximate predictor of MUE. Partial least squares structural equation modeling (GOF = 0.673–0.674; R^2^ = 0.592–0.603) revealed that litter and soil properties had no significant direct effects on CUE or NUE; instead, soil nutrients exerted strong indirect association through a cascade—soil → microbial biomass → enzyme activity—with opposite total effects on CUE (−0.731, *p* < 0.001) versus NUE (+0.755, *p* < 0.001). These findings reveal that the same soil nutrient enrichment that accompanies mixed-species afforestation drives divergent microbial metabolic responses—suppressing CUE while promoting NUE—through a shared cascading structure, with implications for predicting soil carbon and nutrient retention under shifting forest compositions.

## 1. Introduction

Forest soils represent the largest terrestrial organic carbon pool, storing approximately 860 Pg C in the upper 100 cm [1,2]. The persistence and turnover of this vast C pool are fundamentally driven by soil heterotrophic microorganisms [3]. During the decomposition of soil organic matter (SOM), microbes partition assimilated C between biomass synthesis (anabolism) and respiratory energy production (catabolism). This partitioning is quantified as microbial carbon use efficiency (CUE) [4,5]. Given that microbial necromass constitutes a major proportion of stable SOM [6], CUE is increasingly recognized as a master parameter in Earth system models [7]. Even a minor shift in microbial CUE can drastically alter global soil C projections [8,9]. Concurrently, microbial nitrogen and phosphorus use efficiencies (NUE and PUE) regulate nutrient retention and availability, feeding back into plant productivity [10,11]. However, capturing the dynamics of microbial element use efficiencies (MUE) in mixed forests remains challenging due to an incomplete mechanistic understanding of how aboveground vegetation changes regulate belowground microbial physiology.

Forest composition inherently dictates the quantity and quality of plant litter inputs, which serve as the primary energetic and elemental resources for soil decomposers [12,13]. Conventional ecological paradigms postulate that high-quality litter (e.g., low C:N ratio) promotes higher microbial CUE by providing easily assimilable substrates [6,14]. Consequently, mixed-species afforestation is often advocated as a strategy to enhance litter quality and accelerate nutrient cycling [15,16]; however, the effects of mixing are not universally positive. Coniferous species in mixed stands can introduce recalcitrant litter (e.g., lignin-rich pine needles) that may slow decomposition rates and alter microbial community structure [13,14]. The net effect of mixing on soil carbon and nutrient dynamics depends on the specific species combination, litter chemistry, and soil environmental context. However, emerging evidence suggests that the relationship between resource quality and microbial metabolic efficiency is far from linear [17,18]. In addition to litter quality, soil mineral matrices play a critical role in stabilizing organic matter through organo-mineral associations, whereby microbial necromass and decomposition products become protected on mineral surfaces [6,14]. This mineral protection mechanism is now recognized as a primary control on soil organic matter persistence, complementing the biochemical recalcitrance paradigm. The interplay between litter quality, microbial processing, and mineral stabilization ultimately determines the fate of organic carbon in forest soils.

According to ecological stoichiometry theory, microorganisms strive to maintain a relatively stable internal elemental composition despite high variability in resource stoichiometry [10,19]. When the stoichiometric ratio of the available substrate deviates from microbial demand, microbes must dynamically adjust their metabolic fluxes [20,21]. Under nutrient-limiting but C-rich conditions, microbes may lower CUE to “burn off” excess C through overflow metabolism while maximizing NUE to scavenge limiting nutrients [22,23]. Conversely, in highly active, nutrient-rich environments, intense microbial competition and high biomass maintenance costs may force microbes to allocate more C towards synthesizing nutrient-acquiring extracellular enzymes [24,25]. This creates a potential physiological trade-off [26]. Despite its theoretical importance, empirical evidence demonstrating how this trade-off operates across different forest environmental gradients is severely lacking. Traditionally, empirical models have attempted to link CUE and NUE directly to environmental variables like soil pH, temperature, or total organic C [27,28]. However, these direct linkage models often overlook intermediate physiological bottlenecks [29]. Extracellular enzymes, acting as the proximate agents of SOM depolymerization, bridge the gap between environmental nutrient availability and microbial intracellular metabolism [30,31]. We postulate that abiotic environmental factors do not directly determine metabolic efficiencies; rather, their influence is transmitted through indirect biological cascades involving microbial biomass expansion and enzymatic investment [32,33]. Shifts in microbial community composition further mediate these responses, as community structure determines the collective metabolic capabilities of the microbial pool.

The Funiu Mountains, situated at the biogeographic transition between subtropical and warm-temperate zones in central China, harbor a mosaic of forest types that provide an exceptional setting for investigating litter quality effects on microbial metabolism. The co-occurrence of *Larix gmelinii* coniferous plantations, *Quercus aliena* var. *acuteserrata* deciduous broad-leaved forests, and mixed *Q. aliena*–*Pinus armandii* forests within a narrow elevation band minimizes confounding climatic and edaphic variation, enabling a focused assessment of vegetation-driven mechanisms [34]. In this study, we quantified a comprehensive suite of variables across litter chemistry, soil physicochemical properties, microbial biomass, extracellular enzyme activities, and microbial nutrient use efficiencies (CUE, NUE, and PUE) in the three forest types. Using multiple complementary analytical approaches, we aimed to (i) characterize forest type-driven divergence in litter–soil–microbe–enzyme profiles, (ii) examine relationships among CUE, NUE, and PUE across forest types, (iii) identify the primary environmental drivers of MUE, and (iv) evaluate a cascading pathway model linking litter quality to microbial nutrient use efficiency through soil and microbial compartments. These analyses provide a quantitative, pathway-resolved framework for understanding how forest composition shapes microbial metabolic efficiency, with implications for predicting soil carbon and nutrient dynamics under shifting vegetation conditions.

## 2. Materials and Methods

### 2.1. Study Area

This study was conducted at the same experimental sites described in He et al. [34], within the Funiu Mountain National Nature Reserve (33°40′–34°20′ N, 110°0′–112°45′ E), Henan Province, central China. The region lies at the biogeographic transition between the northern subtropical and warm-temperate zones, with a continental monsoon climate (mean annual temperature 12.1–15.1 °C; mean annual precipitation 800–1100 mm). Soils are classified as yellow-brown soil (Haplic Cambisol) developed from granite parent material. Three forest types were selected within a narrow elevation band (1303–1482 m) on northeast-facing slopes: (1) LG: *Larix gmelinii* plantation; (2) QA: *Quercus aliena* var. *acuteserrata* secondary forest; (3) QP: Mixed forest co-dominated by *Quercus aliena* var. *acuteserrata* and *Pinus armandii*. Detailed site characteristics and understory vegetation composition are provided in He et al. [34].

### 2.2. Litter and Soil Sampling

Litter and soil samples were collected from the same 30 plots (10 per forest type) described in He et al. [34] in July 2023 (peak growing season). Briefly, five 10 × 10 m quadrats were established within each forest type. Within each quadrat, five soil cores (4 cm diameter, 0–20 cm depth) and the surface litter layer above each core were collected, composited, and homogenized. This sampling was repeated twice (two parallel replicates) in each quadrat, yielding 10 composite samples per forest type and 30 samples in total. Each composite soil sample was sieved (2 mm) and divided into two portions: one stored at 4 °C for microbial biomass, enzyme activity, and mineral nitrogen analyses, and the other air-dried for physicochemical analyses. A portion of each soil sample was also stored at −80 °C for DNA extraction and subsequent 16S rRNA gene sequencing, as reported in He et al. [34]. Litter samples were oven-dried at 65 °C and ground to 0.25 mm.

### 2.3. Determination of Litter and Soil Properties

Litter cellulose (LC) and lignin (LL) were measured by the acid detergent fiber method [35]. Litter organic carbon (LOC) and soil organic carbon (SOC) were determined by potassium dichromate oxidation; litter total nitrogen (LTN) and soil total nitrogen (STN) by Kjeldahl digestion; and litter total phosphorus (LTP) and soil total phosphorus (STP) by H_2_SO_4_–HClO_4_ digestion followed by molybdenum–antimony colorimetry. Soil water content (SWC) was determined gravimetrically (105 °C, 24 h), soil pH in a 1:2.5 soil–water suspension, and soil bulk density (SBD) by the ring knife method. Soil ammonium nitrogen (SAN) and nitrate nitrogen (SNN) were extracted with 2 M KCl and measured using a continuous flow analyzer (Auto Analyzer 3-AA3, SEAL Analytical, Germany) [36]. Soil available phosphorus (SAP) was extracted with 0.5 M NaHCO_3_ and determined by molybdenum–antimony colorimetry. Litter and soil properties were computed as described in the Table 1 footnotes. These analytical protocols follow those detailed in He et al. [34]. Litter stoichiometric ratios were calculated as: LOC:LTN (LOCN), LC:LTN (LCCN), LL:LTN (LLCN), LOC:LTP (LOCP), LC:LTP (LCCP), LL:LTP (LLCP), LTN:LTP (LNP), and LL:LC (LLC, lignin–cellulose ratio as an index of structural recalcitrance). Soil stoichiometric ratios were calculated as: SOC:STN (SCN), SOC:STP (SCP), and STN:STP (SNP). Microbial biomass carbon (MBC), nitrogen (MBN), and phosphorus (MBP) were determined by the chloroform fumigation–extraction method [36]. Microbial biomass stoichiometric ratios were calculated as MCN = MBC:MBN, MCP = MBC:MBP, and MNP = MBN:MBP. Stoichiometric imbalance ratios were calculated as ImbCN = SCN: MCN, ImbCP = SCP:MCP, ImbNP = SNP:MNP.

### 2.4. Extracellular Enzyme Activities

The potential activities of four extracellular enzymes were assayed using fluorogenic substrates following the high-throughput microplate protocol [37]: β-1,4-glucosidase (BG, EC 3.2.1.21; substrate: 4-MUB-β-D-glucopyranoside; C-acquiring), β-1,4-N-acetylglucosaminidase (NAG, EC 3.2.1.30; substrate: 4-MUB-N-acetyl-β-D-glucosaminide; N-acquiring), L-leucine aminopeptidase (LAP, EC 3.4.11.1; substrate: L-leucine-7-AMC; N-acquiring), and acid phosphatase (ACP, EC 3.1.3.2; substrate: 4-MUB-phosphate; P-acquiring). Enzyme assays were conducted using saturating substrate concentrations (200 µM for MUB-based and AMC-based substrates), following the protocol of Bell et al. [37], to ensure maximum potential enzyme activity. Following the ecoenzymatic stoichiometry framework, enzyme stoichiometric ratios were calculated as follows:

EEAcn = ln(BG)/ln(NAG + LAP)

EEAcp = ln(BG)/ln(ACP)

EEAnp = ln(NAG + LAP)/ln(ACP)

The vector length (VL) and vector angle (VA) of extracellular enzyme activity were calculated to characterize relative carbon versus nutrient limitation:

VL = √[(EEAcn)^2^ + (EEAcp)^2^]

VA = Degrees[atan2(EEAcp, EEAcn)]

A comparatively longer VL suggested a stronger C limitation, whereas VA values below 45° and above 45° reflected relative N and P limitations, respectively.

### 2.5. Calculation of Microbial Nutrient Use Efficiency

Microbial carbon, nitrogen, and phosphorus use efficiencies (CUE, NUE, and PUE) were quantified following the ecoenzymatic stoichiometry framework of Sinsabaugh et al. [10,23]. Dimensionless scalar variables (Sxy) representing relative metabolic allocation to paired resource acquisition were calculated as follows:

Sxy = EEAxy × (Lxy/Bxy)

where EEAxy is the ratio of extracellular enzyme activities for acquiring element x versus element y (e.g., EEAcn = ln(BG)/ln(NAG + LAP) for C vs. N acquisition); Lxy is the ratio of soil total element pools (e.g., LCN = SOC/STN); and Bxy is the ratio of element concentrations in microbial biomass (e.g., BCN = MBC/MBN). The geometric mean of two interacting scalars was used to derive CUE, NUE, and PUE:

CUE = CUEmax × [(SCN × SCP)/((SCN + KCN) × (SCP + KCP))]^0.5

NUE = NUEmax × [(SNC × SNP)/((SNC + KNC) × (SNP + KNP))]^0.5

PUE = PUEmax × [(SPC × SPN)/((SPC + KPC) × (SPN + KPN))]^0.5

where CUEmax, NUEmax, and PUEmax represent theoretical maximum efficiencies, parameterized as 1.0 based on thermodynamic constraints. Half-saturation constants (K) were uniformly set to 0.5, following global-scale validation of established ecoenzyme models.

### 2.6. Statistical Analyses

All statistical analyses were conducted in R version 4.3.1. Before parametric analyses, data were tested for normality (Shapiro–Wilk test) and homogeneity of variance (Levene’s test); variables not meeting assumptions were log- or square-root-transformed as appropriate. One-way ANOVA with forest type as the fixed factor was performed for each indicator, with Tukey’s HSD post hoc test for pairwise comparisons. Effect sizes were quantified as eta-squared (η^2^) and classified as small (η^2^ ≥ 0.01), medium (η^2^ ≥ 0.06), or large (η^2^ ≥ 0.14). Results are presented as mean ± standard error (SE) with different letters denoting significant differences (*p* < 0.05). All F-statistics are reported with degrees of freedom (2, 27). PCoA was constructed using the vegan (version 2.7-3) package which based on Bray–Curtis dissimilarity matrices was conducted separately for four ecological modules prior to any discussion of multivariate patterns: (1) Litter module (14 indicators: LOC, LTN, LTP, LC, LL, LLC, LOCN, LCCN, LLCN, LOCP, LCCP, LLCP, LNP); (2) Soil module (12 indicators: SWC, SBD, pH, SOC, STN, STP, SCN, SCP, SNP, SAN, SNN, SAP); (3) Enzyme module (9 indicators: BG, NAG, LAP, AP, EEAcn, EEAcp, EEAnp, VL, VA); and (4) Biomass module (6 indicators: MBC, MBN, MBP, MCN, MCP, MNP). Variables were z-score standardized prior to ordination. Permutational multivariate analysis of variance (Adonis) with 999 permutations tested forest type effects; 95% confidence ellipses were overlaid on ordination plots. Random forest models were constructed to identify the primary predictors of CUE, NUE, and PUE across all environmental variables using the randomForest (version 4.7-1.2) and rfPermute (version 2.5.5) packages [38]. Two PLS-SEM models (CUE and NUE as target variables) were constructed using the plspm (version 0.6.0) package. Model fit was assessed using the goodness-of-fit (GOF) index [39]. Standardized path coefficients (direct, indirect, and total effects) were estimated via the partial least squares algorithm with bootstrapping (n = 1000). Prior to PLS-SEM analyses, variance inflation factors (VIF) were calculated for all predictor variables. Variables with VIF > 5 were sequentially removed to reduce multicollinearity. The package of ggplot2 (version 4.0.3) was used for visualization.

## 3. Results

### 3.1. Forest Types Drive Multidimensional Differentiation of Litter, Soil, and Microbial Properties

**Figure 1:**
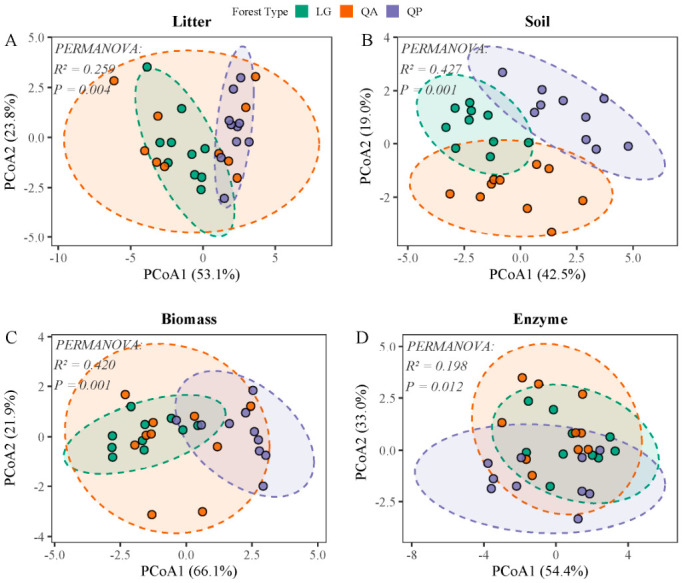
Principal coordinate analysis (PCoA) based on Bray−Curtis dissimilarity matrices for four ecological modules across three forest types. (**A**) Litter module (14 indicators); (**B**) Soil module (12 indicators); (**C**) Microbial biomass module (6 indicators); (**D**) Enzyme module (9 indicators). Each point represents one composite sample (n = 10 per forest type). Ellipses indicate 95% confidence intervals. PERMANOVA R^2^ and *p*-values are shown for each module. LG: *Larix gmelinii* plantation; QA: *Quercus aliena* var. *acuteserrata* secondary forest; QP: mixed *Quercus aliena* var. *acutiserrata* and *Pinus armandii* forest.

Principal coordinate analysis (PCoA) based on Bray–Curtis dissimilarity matrices revealed distinct multivariate differentiation among the three forest types across all four ecological modules (Figure 1). PERMANOVA confirmed highly significant effects of forest type on the litter (R^2^ = 0.259, *p* = 0.004), soil (R^2^ = 0.427, *p* = 0.001), microbial biomass (R^2^ = 0.420, *p* = 0.001), and enzyme modules (R^2^ = 0.198, *p* = 0.012). Notably, the soil and microbial biomass modules exhibited the strongest structural differentiation (highest R^2^ values), indicating that forest type exerted profound effects on the belowground edaphic environment. Univariate analyses further elucidated specific variations within these modules (Table 1). In terms of litter chemistry, the mixed forest (QP) produced litter with significantly higher organic carbon (LOC) and total nitrogen (LTN) concentrations compared to the two pure forests (LG and QA) (*p* = 0.005). The cellulose content (LC) was also highest in QP. Conversely, key indicators of litter recalcitrance, such as the lignin-to-cellulose (LLC) and lignin-to-nitrogen (LLCN) ratios, were significantly lower in QP relative to LG (*p* = 0.016). Litter total phosphorus (LTP) remained stable across all forest types (*p* = 0.120). Consistent with the aboveground inputs, belowground soil physicochemical properties varied significantly among forest types (Table 1). The QP treatment accumulated significantly larger carbon and nitrogen pools, with SOC and STN concentrations being 1.70–2.35 times and 1.41–2.19 times higher than those in QA and LG, respectively (*p* < 0.001, η^2^ > 0.46). However, soil total phosphorus (STP) exhibited no significant inter-group differences (*p* = 0.706). Regarding nutrient availability, available ammonium (SAN) was significantly enriched in QA and QP relative to LG (*p* = 0.002), whereas available phosphorus (SAP) showed the opposite trend, being significantly lower in QA and QP than in LG (*p* < 0.001). Physical parameters also varied: soil water content (SWC) peaked in QA (*p* < 0.001), and soil bulk density (SBD) slightly increased along the LG-QA-QP gradient (*p* = 0.005). Soil pH showed no significant variation. Microbial biomass pools exhibited trends similar to those of soil resources. Both MBC and MBN were highest in the QP forest, significantly exceeding those in LG and QA (*p* < 0.001). Similar to LTP and STP, microbial biomass phosphorus (MBP) was unaffected by forest type (*p* = 0.086).

### 3.2. Soil and Microbial Stoichiometric Traits and Their Imbalances Across Forest Types

**Figure 2:**
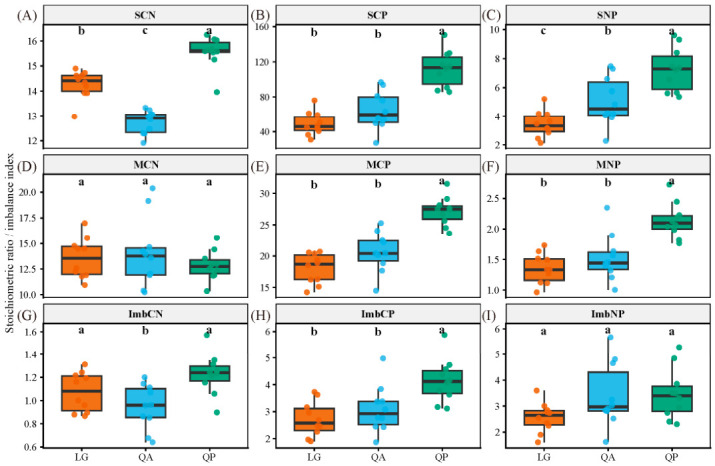
Soil chemical stoichiometry (**A**–**C**), microbial biomass stoichiometry (**D**–**F**), and stoichiometric imbalance (**G**–**I**) across three forest types in the Funiu Mountains. Bars represent means ± SE (n = 10 per forest type). Different lowercase letters above bars indicate significant differences among forest types (Tukey’s HSD, *p* < 0.05). SCN, SOC:STN; SCP, SOC:STP; SNP, STN:STP; MCN, MBC:MBN; MCP, MBC:MBP; MNP, MBN:MBP; ImbCN, SCN:MCN; ImbCP, SCP:MCP; ImbNP, SNP:MNP.

Soil ecological stoichiometry was significantly altered by forest types (Figure 2A–C). The QP exhibited significantly higher SCN and SCP ratios compared to both pure forests (*p* < 0.05). Specifically, SCN followed a distinct order of QP > LG > QA, while SCP was highest in QP, with no significant difference observed between LG and QA. Additionally, the SNP increased progressively and significantly along the gradient from LG to QA to QP (all *p* < 0.05). In contrast to the highly variable soil nutrient stoichiometry, microbial biomass C:N:P ratios demonstrated constrained variation (Figure 2D–F). MCN remained statistically invariant across all three forest types (*p* > 0.05), consistent with the core principles of ecological stoichiometry. However, the MCP and MNP ratios were not strictly constrained; both were significantly elevated in the QP treatment compared to the LG and QA treatments (*p* < 0.05). Despite this treatment-induced variation in P-related microbial ratios, the magnitude of their shifts was notably constrained compared to the profound divergence observed in the corresponding soil ratios. The disparity between soil resource supply and microbial demand generated specific patterns of stoichiometric imbalance (Figure 2G–I). The C:P imbalance (ImbCP) was significantly exacerbated in the QP forest compared to LG and QA (*p* < 0.05), aligning closely with the elevated SCP in this treatment. The N:P imbalance (ImbNP) showed a trend toward higher values in QA and QP compared to LG, but this difference did not reach statistical significance (*p* > 0.05). Interestingly, the C:N imbalance (ImbCN) did not exhibit a linear trend across the forest types; instead, it was significantly lower in the QA treatment compared to both LG and QP (*p* < 0.05), while no significant difference was detected between the latter two.

### 3.3. Soil Extracellular Enzyme Activities and Microbial Nutrient Limitation

**Figure 3:**
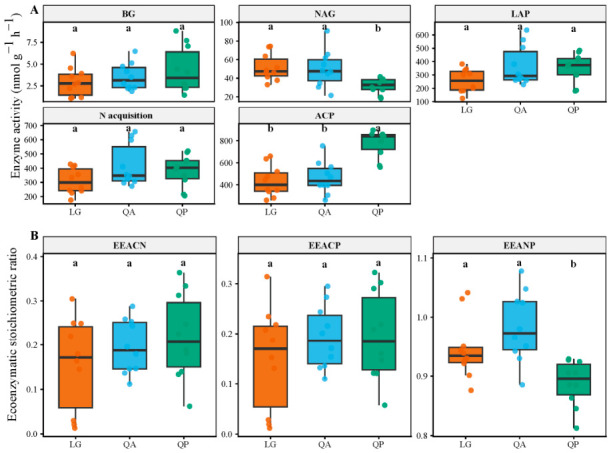
Extracellular enzyme activities and enzyme stoichiometric ratios across three forest types in the Funiu Mountains. (**A**) Enzyme activities: BG, β-1,4-glucosidase; NAG, β-1,4-N-acetylglucosaminidase; LAP, L-leucine aminopeptidase; ACP, acid phosphatase. (**B**) Enzyme stoichiometric ratios: EEAcn, EEAcp, EEAnp. Bars represent means ± SE (n = 10 per forest type). Different lowercase letters indicate significant differences (Tukey’s HSD, *p* < 0.05).

Soil extracellular enzyme activities exhibited distinct, enzyme-specific responses to the forest types (Figure 3A). Acid phosphatase (ACP) activity, representing microbial phosphorus acquisition, was significantly elevated in the mixed forest (QP) compared to the pure forests (LG and QA) (*p* < 0.05). Conversely, the activity of *β*-1,4-N-acetylglucosaminidase (NAG), a primary nitrogen-acquiring enzyme, was significantly suppressed in the QP treatment relative to LG and QA (*p* < 0.05). The activities of *β*-1,4-glucosidase (BG) and L-leucine aminopeptidase (LAP), as well as the combined N-acquisition activity (NAG + LAP), remained relatively stable, with no statistically significant differences observed among the three forest types (all *p* > 0.05). Enzymatic stoichiometric ratios further reflected these shifts in microbial resource acquisition strategies (Figure 3B). While the EEACN and EEACP ratios showed no significant inter-group differences (*p* > 0.05), the EEANP was significantly altered by forest type (*p* < 0.05). Specifically, EEANP was significantly lower in the QP forest compared to both LG and QA, with no significant difference detected between the latter two pure forests.

**Figure 4:**
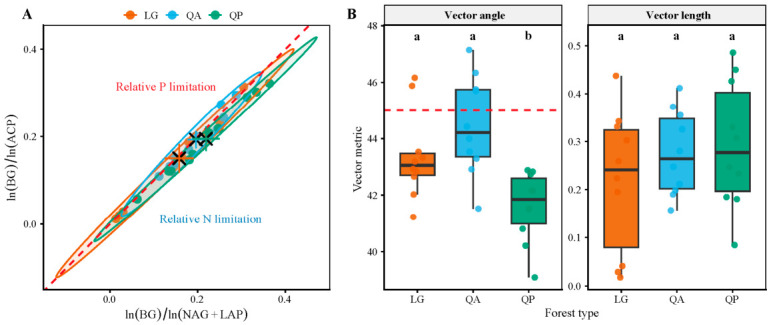
Microbial nutrient limitation status assessed by enzyme stoichiometric vector analysis. (**A**) Scatter plot of ecoenzymatic stoichiometry showing the relative position of each sample relative to the 1:1 reference line (red line); points below the line indicate relative N limitation, points above indicate relative P limitation. (**B**) Vector angles (VA) and Vector length (VL) across three forest types; VA < 45° indicates relative N limitation; VA > 45° indicates relative P limitation. The larger the value of VL, the stronger the carbon limitation. Bars represent means ± SE (n = 10 per forest type). Different lowercase letters indicate significant differences (Tukey’s HSD, *p* < 0.05).

Vector analysis of the enzymatic stoichiometry provided an integrated assessment of microbial relative nutrient limitation (Figure 4). Across all forest types, the data points in the scatter plot predominantly clustered below the 1:1 reference line (Figure 4A), and all calculated vector angles (VA) were consistently below the 45° threshold (Figure 4B). This pattern indicates a pervasive state of relative microbial nitrogen limitation across the entire study system. Furthermore, the severity of this relative limitation varied significantly among forest types. The QP forest exhibited a significantly lower VA compared to both LG and QA (*p* < 0.05), indicating that the microbial community in the mixed forest experienced the most acute relative nitrogen limitation. In contrast, the vector length (VL), a proxy for relative carbon limitation, ranged from 0.22 to 0.29 and showed no significant differences among the three forest types, indicating a consistent level of microbial carbon demand across the gradient.

### 3.4. Microbial Carbon and Nutrient Use Efficiencies and Their Trade-Offs

**Figure 5:**
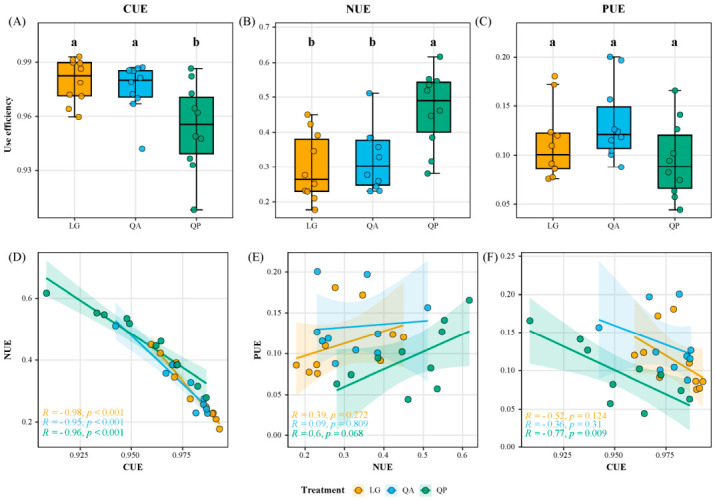
Pairwise relationships among microbial carbon use efficiency (CUE), nitrogen use efficiency (NUE), and phosphorus use efficiency (PUE) across three forest types. (**A**–**C**) Box plots of CUE, NUE, and PUE by forest type. (**D**–**F**) Bivariate scatter plots of CUE vs. NUE, NUE vs. PUE, and CUE vs. PUE. Each point represents one composite sample (n = 10 per forest type). The shaded areas represents 95% confidence interval. Different lowercase letters indicate significant differences (Tukey’s HSD, *p* < 0.05).

Microbial elemental use efficiencies exhibited divergent responses to the different forest types (Figure 5A–C). Carbon use efficiency (CUE) was significantly lower in the mixed forest (QP) compared to the pure forests (LG and QA) (*p* < 0.05). In stark contrast, nitrogen use efficiency (NUE) followed the opposite trend, being significantly elevated in the QP treatment relative to both LG and QA (*p* < 0.05). Phosphorus use efficiency (PUE) remained relatively stable, with no statistically significant differences detected among the three forest types (*p* > 0.05). Bivariate relationship analyses revealed a strict and robust trade-off between microbial C and N use efficiencies across all forest types (Figure 5D). CUE and NUE exhibited highly significant negative correlations within each individual forest stand (LG: R = −0.98; QA: *R* = −0.95; QP: *R* = −0.96; all *p* < 0.001). Notably, the data points for the QP treatment distinctly clustered in the high-NUE/low-CUE quadrant, whereas the LG and QA treatments clustered in the low-NUE/high-CUE region, reflecting a systematic shift in metabolic strategies driven by forest type. Conversely, relationships involving PUE were highly variable and generally lacked consistent linear patterns across all forest types (Figure 5E,F). The correlation between NUE and PUE (Figure 5E) was not significant for either the LG (*p* = 0.272) or QA (*p* = 0.809) forests, with only a marginally positive trend observed in the QP forest (R = 0.60, *p* = 0.068). Similarly, the relationship between CUE and PUE (Figure 5F) was non-significant in the LG and QA treatments (*p* > 0.05), but demonstrated a significant negative correlation specifically within the QP mixed forest (R = −0.77, *p* = 0.009).

### 3.5. Key Drivers and Regulatory Pathways of Microbial Metabolic Efficiencies

**Figure 6:**
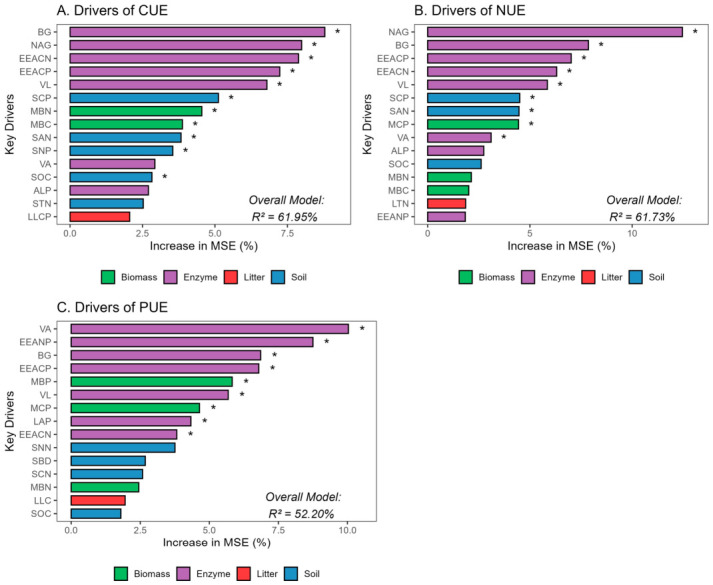
Random forest analysis of environmental drivers of microbial nutrient use efficiency. Variable importance (%IncMSE) for (**A**) CUE (pseudo-R^2^ = 61.95%), (**B**) NUE (pseudo-R^2^ = 61.73%), and (**C**) PUE (pseudo-R^2^ = 52.20%). Predictors are color-coded by category: red, Litter; blue, Soil; purple, Enzyme; green, Biomass. Significance levels assessed by 1000 permutations (* *p* < 0.05).

Random forest models identified the relative importance of aboveground, edaphic, and microbial variables in predicting elemental use efficiencies, explaining 62.0%, 61.7%, and 52.2% of the variance in CUE, NUE, and PUE, respectively (Figure 6). A highly consistent pattern emerged for both CUE and NUE: variables within the “Enzyme” category overwhelmingly dominated the prediction. Specifically, the top five drivers for CUE (BG, NAG, EEACN, EEACP, and VL) and NUE (NAG, BG, EEACP, EEACN, and VL) were exclusively extracellular enzyme-related parameters. In contrast, “Litter” properties consistently ranked at the bottom of variable importance across all models. PUE exhibited a slightly more distributed importance profile, though enzyme-related variables (VA, EEANP) remained the most critical predictors.

**Figure 7:**
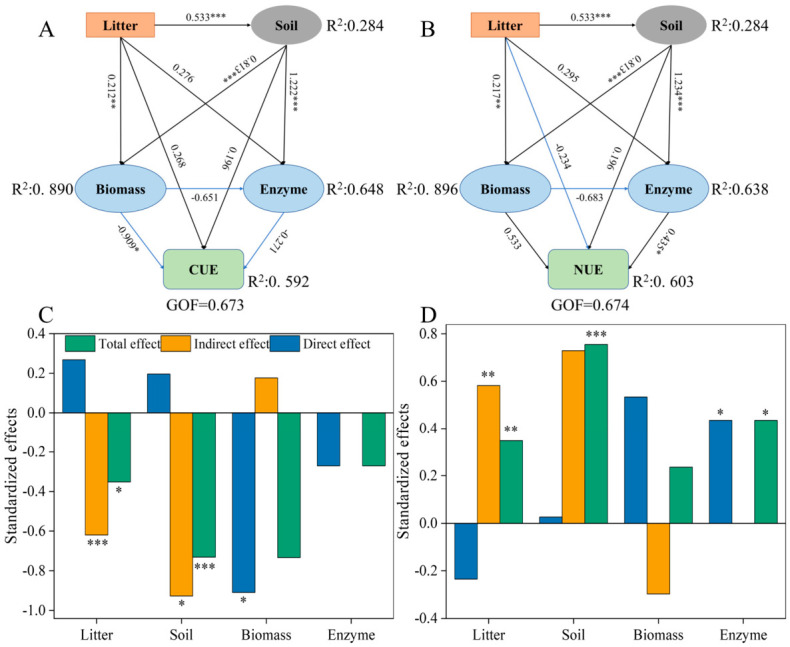
Partial least squares structural equation model (PLS-SEM) of the cascading pathway from litter quality to microbial nutrient use efficiency. (**A**) CUE model (GOF = 0.673, R^2^ = 0.592). (**B**) NUE model (GOF = 0.674, R^2^ = 0.603). (**C**) Standardized total, indirect, and direct effects for the CUE model. (**D**) Standardized total, indirect, and direct effects for the NUE model. Black arrows: positive standardized path coefficients; blue arrows: negative standardized path coefficients. *: *p* < 0.05; **: *p* < 0.01; ***: *p* < 0.001; Values on arrows are standardized path coefficients (β). R^2^ values are shown within endogenous construct boxes. GOF, goodness of fit index.

To further disentangle the cascading mechanisms linking litter inputs to these metabolic outcomes, partial least squares structural equation modeling (PLS-SEM) was constructed (Figure 7). The models yielded high goodness-of-fit values (GOF > 0.67) and explained 59.2% and 60.3% of the variance in CUE and NUE, respectively. A fundamental structural cascade was identified: Litter quality significantly altered soil physicochemical properties (β = 0.533, *p* < 0.001), which in turn strongly expanded both microbial biomass (β > 0.81, *p* < 0.001) and Enzyme activities (β > 1.22, *p* < 0.001). Crucially, neither Litter nor soil exerted any significant direct effects on either CUE or NUE (all direct paths *p* > 0.05; Figure 7A,B). Their influences were entirely mediated through indirect biological pathways, ultimately generating highly asymmetric total effects on CUE versus NUE (Figure 7C,D). For CUE, microbial Biomass exerted a significant negative direct effect (β = −0.909, *p* < 0.05). Mediated primarily through this downstream biological node, the overall cascade resulted in significant negative total effects of both Litter (βtotal = −0.352, *p* < 0.01) and soil (βtotal = −0.731, *p* < 0.001) on carbon use efficiency. Conversely, the regulatory pathways for NUE exhibited an opposing pattern. Enzyme activity, rather than Biomass, exerted a significant positive direct effect on NUE (β = 0.435, *p* < 0.05). Consequently, the overall cascade generated significant positive total effects of Litter (βtotal = 0.348, *p* < 0.01) and soil (βtotal = 0.755, *p* < 0.001) on nitrogen use efficiency. This divergence at the terminal biological nodes (Biomass suppressing CUE vs. Enzyme promoting NUE) structurally accounts for the divergent responses of these two efficiencies to the environmental gradients.

## 4. Discussion

### 4.1. High-Quality Litter in Mixed Forests Drives the Expansion of Belowground Resource Pools

Our multidimensional analysis revealed that forest types profoundly orchestrate the differentiation of edaphic environments, primarily initiated by variations in aboveground litter inputs. The mixed forest (QP) produced litter characterized by higher lability (lower lignin-to-cellulose and lignin-to-nitrogen ratios) and enriched nutrients (higher LOC and LTN). According to the Microbial Efficiency-Matrix Stabilization (MEMS) framework, such high-quality, labile plant inputs can efficiently accelerate organic matter decomposition and incorporation into soil structural pools [6,13]. Consequently, we observed a massive expansion of soil carbon (SOC) and nitrogen (STN) pools in the QP forest, which were up to 2.35 times higher than those in pure stands. This aboveground resource enrichment subsequently alleviated environmental constraints on microbial growth, leading to significantly expanded microbial biomass (MBC and MBN) in the mixed forest. These findings align with previous observations that tree species mixing and higher plant diversity can enhance belowground biogeochemical cycling and stimulate microbial anabolism, thereby driving soil organic carbon accumulation [7,15,16]. However, the mechanisms underlying SOC accumulation in mixed forests likely extend beyond simple litter decomposition [40]. The high-quality litter inputs in QP may have promoted rapid microbial processing and necromass formation, which subsequently becomes stabilized on mineral surfaces through organo-mineral associations [6,14,41]. This mineral protection mechanism is now recognized as a primary control on SOM persistence, complementing the biochemical recalcitrance paradigm [31]. In the Funiu Mountain system, the granitic parent material provides reactive mineral surfaces that may facilitate such stabilization, potentially explaining the paradox of high SOC accumulation despite relatively low microbial CUE in QP.

It should be noted that the positive effect of mixed-species stands on belowground C accumulation is not universal. In European temperate forests, Prescott and Vesterdal [13] showed that coniferous components in mixed stands can introduce recalcitrant litter that slows decomposition. Similarly, in subtropical Chinese forests, Wang et al. [17] found that the effect of mixing on soil microbial activity depends on the specific species combination and litter chemistry. In our system, the *Pinus armandii* component in QP contributes pine needle litter that is lignin-rich, yet the overall mixed litter quality (as indicated by lower LLC and LLCN ratios) remains higher than that of pure conifer stands, suggesting that the broadleaf component ameliorates the recalcitrance effect. This context-dependency underscores the importance of species-specific litter chemistry in determining the net effect of forest mixing on belowground processes.

### 4.2. Strict Microbial Stoichiometric Homeostasis Exacerbates Resource Imbalances

Despite the highly variable soil nutrient stoichiometry (SCN, SCP, and SNP ratios) driven by different forest types, the microbial communities exhibited constrained variation, particularly in the MCN ratio. The constrained variation of MCN is consistent with the core principles of ecological stoichiometry, which posit that microbial biomass maintains a relatively rigid elemental composition compared to fluctuating environmental resources [19,20]. While MCP and MNP showed some flexibility, being significantly elevated in QP, their shifts were substantially constrained compared to the profound divergence observed in soil resources. This fundamental discrepancy between fluctuating resource supply and constrained microbial demand inherently generates stoichiometric imbalances [11]. In our study, the accelerated accumulation of SOC relative to P in the mixed forest, coupled with constrained microbial stoichiometry, severely exacerbated the C:P imbalances (ImbCP was significantly higher in QP than in LG and QA; Figure 2G). The N:P imbalance (ImbNP) showed a similar trend but did not reach statistical significance (*p* > 0.05), likely due to high variability in MNP across plots. This indicates that while mixed forests are carbon- and nitrogen-rich, they create a stoichiometrically decoupled environment. Microbes inhabiting such environments are compelled to adjust their physiological and metabolic strategies to cope with these disproportionate resource availabilities [21,42].

### 4.3. Enzymatic Adaptation Reveals Intensified Relative Nitrogen Limitation

Vector analysis of enzymatic stoichiometry provided a counterintuitive yet mechanistically sound insight into microbial nutrient limitation. Despite the absolute enrichment of total and available nitrogen (STN and SAN) in the QP forest, the microbial community in this mixed stand experienced the most acute relative nitrogen limitation (indicated by the lowest Vector Angle) [31,32]. This apparent paradox can be explained by the synthesis of stoichiometric imbalance and biological expansion. The massive accumulation of highly available carbon (SOC) and the subsequent surge in microbial biomass in the mixed forest dramatically amplified the aggregate biological demand for nitrogen [22,33]. This explosive anabolic demand outpaced the supply, shifting the community into a severe relative N-deficit state. To adapt, microbes dynamically modulated their extracellular enzyme production [30]. Interestingly, classical N-acquiring enzymes (NAG) were specifically suppressed in the QP treatment. This suppression might reflect a shift in microbial resource acquisition strategies: rather than synthesizing costly NAG to degrade complex organic N, microbes might preferentially assimilate the highly abundant available ammonium (SAN) present in the QP soil, thereby optimizing their energy investment under relative N limitation [22,43]. This enzyme-level adaptation is consistent with the bacterial community niche breadth data reported by He et al. [34], who found that the bacterial community in QP exhibited narrower niche breadth compared to pure forests, suggesting greater specialization in resource utilization. Such specialization may reflect community-level adaptation to the stoichiometrically imbalanced environment, where specific taxa capable of efficient N acquisition under P-limited conditions are selected for. Comparable patterns of enzyme adaptation under nutrient imbalance have been reported in temperate grasslands [23] and subtropical forests [17], where relative N limitation driven by elevated C inputs similarly triggered down-regulation of NAG activity.

### 4.4. Biological Cascades Fundamentally Decouple CUE and NUE Trade-Offs

A core finding of this study is the strict, highly significant negative trade-off between CUE and NUE across all forest types, with the mixed forest distinctly clustering in the low-CUE/high-NUE quadrant. This robust trade-off reflects a fundamental thermodynamic and stoichiometric constraint on microbial metabolism [4,10,23]. Crucially, our PLS-SEM and Random Forest models disentangled the regulatory mechanisms driving these metabolic shifts, revealing that environmental factors (litter and soil) do not directly determine metabolic efficiencies. Instead, they are associated with these efficiencies through a divergent biological pathway. On one hand, the enriched soil resources in the QP forest were associated with larger microbial biomass, which showed a negative direct association with CUE (β = −0.909). This phenomenon is likely attributable to the higher maintenance respiration costs and intensified internal competition associated with sustaining a larger biomass pool, which diverts carbon away from growth (anabolism) towards energy production [17,44]. On the other hand, extracellular enzymes emerged as the direct positive regulators of NUE (β = 0.435). Associated with the acute relative N limitation in the mixed forest, enzymatic adjustments were systematically positively associated with NUE to maximize the retention of this limiting element [24,33]. This structural divergence at the terminal biological nodes—where biomass expansion shows negative association with CUE while enzymatic adaptation shows positive association with NUE. While our PLS-SEM analysis indicates that microbial biomass is significantly associated with CUE variation, we acknowledge that other mechanisms may also contribute. First, shifts in the bacterial-to-fungal ratio could influence CUE, as fungi generally have higher CUE than bacteria [16]. He et al. [34] showed that the bacterial community in QP is distinct from that in pure forests, with lower network complexity but higher modularity, suggesting a more compartmentalized community structure. If the fungal community shows parallel shifts, the altered bacterial-to-fungal ratio could partially explain the lower CUE in QP. Unfortunately, fungal community data are not available in the present study, representing a limitation. Second, the quality of organic matter—particularly the proportion of labile vs. recalcitrant C pools—may directly affect CUE independently of total biomass. Future studies incorporating fungal community composition and detailed organic matter fractionation are needed to disentangle these alternative hypotheses.

PUE showed no significant forest-type effect (*p* > 0.05), suggesting that the metabolic trade-off is specific to the C–N axis rather than a generalized response. This element-specific decoupling is consistent with the ecoenzymatic stoichiometry framework, where PUE is buffered by the relatively stable soil P observed across forest types (STP, F(2,27) = 0.35, *p* = 0.706). The retention of PUE in the analysis is justified by the need for conceptual completeness within the C–N–P trinity of the ecoenzymatic stoichiometry framework [10,23], and by the fact that He et al. [34] identified microbial biomass C:P (MCP) as a significant driver of bacterial community assembly in these forests, indicating that P-related microbial processes remain ecologically relevant despite the non-significant PUE response.

### 4.5. Limitations and Practical Implications

Several limitations of this study should be acknowledged. First, sampling was conducted at a single time point (July 2023, peak growing season). Soil microbial communities, enzyme activities, and nutrient use efficiencies exhibit strong seasonal variation [23,30]. Our results thus represent a snapshot of peak-season conditions and may not capture the full range of seasonal variability. Future studies should incorporate multiple seasonal sampling campaigns to assess the temporal stability of the observed patterns. Second, the ratio of 41 variables to 30 observations exceeds the recommended variable-to-observation ratio of 1:5 to 1:10 for multivariate analyses. Although VIF pre-filtering was performed to reduce multicollinearity, and the Random Forest and PLS-SEM results were cross-validated via bootstrapping (n = 1000), we acknowledge that the high variable-to-sample ratio may inflate the risk of Type I errors. Future studies with larger sample sizes are needed to confirm the robustness of our findings. Third, while He et al. [34] provided bacterial community composition data for the same sites, fungal community data are not available. As fungi generally have higher CUE than bacteria [16] and play distinct roles in organic matter decomposition, the absence of fungal data limits our ability to fully evaluate the alternative hypothesis that bacterial-to-fungal ratio shifts drive CUE variation. Future studies should incorporate both bacterial and fungal community composition data. Fourth, PLS-SEM tests the consistency of data with a hypothesized model structure but does not establish causation. The same dataset was used to both identify and evaluate the model. Experimental manipulations (e.g., litter addition/removal, enzyme inhibition experiments) and independent validation datasets are needed to confirm the causal pathways proposed in this study.

From a forest management perspective, our findings suggest that the choice of forest type involves trade-offs between different ecosystem functions. The mixed forest (QP) accumulates significantly more soil organic carbon and total nitrogen than pure forests, making mixed-species stands preferable for maximizing total soil C and N stocks in this region. However, the lower microbial CUE in mixed forests implies that a larger proportion of C inputs may be lost as CO_2_, meaning that the carbon sequestration efficiency per unit input is lower. For carbon sequestration objectives, mixed stands of broadleaf and conifer species appear advantageous in terms of total C accumulation, but the long-term stability of this stored carbon warrants further investigation. For nutrient retention objectives, the higher NUE in mixed forests suggests better nitrogen conservation. These recommendations are specific to the Funiu Mountain region and the species combinations studied; extrapolation to other regions should be made with caution.

## 5. Conclusions

This study reveals how forest compositional differences in the Funiu Mountains relate to soil microbial metabolic strategies. In the mixed forest (QP), high-quality litter inputs are associated with expanded soil C and N pools but severe stoichiometric imbalance. Microbial communities exhibit constrained biomass stoichiometry, and enzyme production adjusts to cope with relative N limitation. These adjustments are associated with divergent CUE and NUE—lower CUE but higher NUE in the mixed forest—reflecting a metabolic trade-off. Environmental variables influence MUE indirectly through biological cascades (biomass and enzyme activity) rather than directly. These findings suggest that Earth system models could benefit from incorporating indirect biological pathways and stoichiometric trade-offs when predicting soil C and nutrient dynamics under shifting forest composition. Future research should validate these associational patterns with experimental manipulations and extend the framework to include fungal communities.

## Figures and Tables

**Table 1:** Litter chemistry, soil physicochemical and microbial biomass properties across different forest types (Mean ± SE).

Category	Indicator	LG	QA	QP	F(2,27)	*p*	η^2^
Litter	LOC (g/kg)	286.45 ± 14.09 b	294.46 ± 34.44 b	393.89 ± 7.75 a	7.44	0.003	0.355
	LTN (g/kg)	14.61 ± 0.89 b	14.83 ± 1.35 b	18.95 ± 0.35 a	6.60	0.005	0.328
	LTP (g/kg)	0.75 ± 0.03 a	0.66 ± 0.05 a	0.76 ± 0.02 a	2.29	0.120	0.145
	LC (g/kg)	213.52 ± 6.20 b	203.49 ± 14.75 b	253.41 ± 8.08 a	6.51	0.005	0.325
	LL (g/kg)	373.70 ± 10.29 a	320.21 ± 3.35 b	347.29 ± 7.58 a	12.30	<0.001	0.477
	LLC	1.75 ± 0.03 a	1.65 ± 0.12 ab	1.39 ± 0.06 b	5.67	0.009	0.296
	LOCN	19.49 ± 0.44 a	19.49 ± 0.74 a	20.85 ± 0.57 a	1.74	0.194	0.114
	LCCN	15.19 ± 1.21 a	14.14 ± 0.76 a	13.46 ± 0.62 a	0.94	0.402	0.065
	LLCN	26.49 ± 1.90 a	23.61 ± 2.58 ab	18.37 ± 0.49 b	4.83	0.016	0.264
	LOCP	379.01 ± 14.81 b	436.14 ± 35.13 ab	519.81 ± 18.81 a	8.33	0.002	0.381
	LCCP	289.81 ± 14.74 a	310.50 ± 17.67 a	335.39 ± 16.75 a	1.93	0.165	0.125
	LLCP	504.95 ± 18.95 a	504.52 ± 36.57 a	458.95 ± 18.90 a	1.02	0.374	0.070
	LNP	19.55 ± 0.90 b	22.12 ± 1.01 ab	24.94 ± 0.66 a	9.59	<0.001	0.415
Soil	SWC	13.09 ± 1.04 b	21.23 ± 0.90 a	13.16 ± 0.95 b	23.49	<0.001	0.635
	SBD	0.80 ± 0.02 b	0.85 ± 0.02 ab	0.89 ± 0.02 a	6.54	0.005	0.326
	pH	4.97 ± 0.08 a	5.20 ± 0.11 a	4.94 ± 0.07 a	2.49	0.102	0.156
	SOC (g/kg)	11.42 ± 0.91 b	15.85 ± 1.86 b	26.87 ± 2.50 a	18.04	<0.001	0.572
	STN (g/kg)	0.80 ± 0.06 b	1.24 ± 0.14 b	1.75 ± 0.18 a	11.53	<0.001	0.461
	STP (g/kg)	0.24 ± 0.01 a	0.25 ± 0.01 a	0.24 ± 0.01 a	0.35	0.706	0.025
	SAN (mg/kg)	17.43 ± 1.76 b	29.99 ± 3.15 a	29.29 ± 2.35 a	8.07	0.002	0.374
	SNN (mg/kg)	0.90 ± 0.17 a	0.91 ± 0.11 a	0.68 ± 0.17 a	0.67	0.519	0.047
	SAP (mg/kg)	11.97 ± 2.20 a	3.74 ± 1.06 b	4.11 ± 0.32 b	10.68	<0.001	0.442
Biomass	MBC (mg/kg)	158.31 ± 12.98 b	207.83 ± 22.65 b	304.88 ± 21.93 a	14.35	<0.001	0.515
	MBN (mg/kg)	12.08 ± 1.36 b	15.28 ± 1.95 b	23.75 ± 1.44 a	14.11	<0.001	0.511
	MBP (mg/kg)	8.78 ± 0.60 a	9.95 ± 0.84 a	11.19 ± 0.75 a	2.70	0.086	0.166

Note: Different letters indicate significant differences at *p* < 0.05 based on Tukey’s HSD test. Effect sizes are presented as eta squared (η^2^). F-values are reported with degrees of freedom (2, 27) for all one-way ANOVAs. LG: *Larix gmelinii* plantation; QA: *Quercus aliena* var. *acuteserrata* secondary forest; QP: mixed *Q. aliena* var. *acuteserrata* + *Pinus armandii* forest. Abbreviations: LOC, litter organic carbon; LTN, litter total nitrogen; LTP, litter total phosphorus; LC, litter cellulose; LL, litter lignin; LLC, LL:LC; LOCN, LOC:LTN; LCCN, LC:LTN; LLCN, LL:LTN; LOCP, LOC:LTP; LCCP, LC:LTP; LLCP, LL:LTP; LNP, LTN:LTP; SWC, soil water content; SBD, soil bulk density; SOC, soil organic carbon; STN, soil total nitrogen; STP, soil total phosphorus; SAN, soil ammonium nitrogen; SNN, soil nitrate nitrogen; SAP, soil available phosphorus; MBC, microbial biomass carbon; MBN, microbial biomass nitrogen; MBP, microbial biomass phosphorus.

## Data Availability

The original data generated in this study are included in this article. Further enquiries can be directed to the corresponding author.
